# Efficacy of a *Coriolus* *versicolor*-Based Vaginal Gel in Human Papillomavirus-Positive Women Older Than 40 Years: A Sub-Analysis of PALOMA Study

**DOI:** 10.3390/jpm12101559

**Published:** 2022-09-22

**Authors:** Santiago Palacios Gil-Antuñano, Luis Serrano Cogollor, Andrés C. López Díaz, Silvia P. González Rodríguez, Damián Dexeus Carter, Cristina Centeno Mediavilla, Pluvio Coronado Martín, Jesús de la Fuente Valero, José A. López Fernández, Cristina Vanrell Barbat, Javier Cortés Bordoy

**Affiliations:** 1 Instituto Palacios, Salud y Medicina de la Mujer, C. Antonio Acuña, 9, 28009 Madrid, Spain; 2 Obstetrics and Gynaecology Unit, HM Gabinete Velázquez, C. de Jorge Juan, 19, 1°, 28001 Madrid, Spain; 3 Obstetrics and Gynaecology Department, Hospital Quironsalud, Av. Imperio Argentina, 1, 29004 Málaga, Spain; 4 Clínica Ginecológica Women’s CD, C. Gironella, 6, Planta 2 (Edificio 3), 08017 Barcelona, Spain; 5 Clínica Diatros, Crta. de Santa Creu de Calafell, 100, 08850 Gavà, Spain; 6 Gynecological Oncology Unit, Hospital Clínico San Carlos, C. del Prof Martín Lagos, 28040 Madrid, Spain; 7 Obstetrics and Gynaecology Department, Hospital Universitario Infanta Leonor, Av. Gran Vía del Este, 80, 28031 Madrid, Spain; 8 Obstetrics and Gynaecology Department, Hospital General Universitario, C. Pintor Baeza, 11, 03010 Alicante, Spain; 9 STI Unit, Hospital de la Santa Creu i Sant Pau, C. Sant Antoni Maria Claret, 167, 08025 Barcelona, Spain; 10 Private Practice, C. Alfonso el Magnánimo, 29, 07004 Palma, Spain

**Keywords:** *Coriolus versicolor*, human papillomavirus, cervical lesions, clearance, high risk, vaginal gel, age

## Abstract

In the PALOMA trial, Papilocare^®^ demonstrated efficacy in repairing low-grade cervical lesions related to human papillomavirus (HPV). This sub-analysis aimed to evaluate its efficacy in repairing these cervical lesions and clearing HPV in women aged older than 40 years. This was a multicenter, randomized, open-label, parallel-group, controlled clinical trial. Patients with low-degree HPV-dependent cervical lesions receiving 6-month treatment with the vaginal gel were compared to those with a watchful waiting approach. Among the 41 women analyzed (aged 47.7 years), 31 presented high-risk (HR) oncogenic HPV subtypes, and 14 had 16-18-31 HPV genotypes. After 6 months, normalized cytology and concordant colposcopy were achieved by a greater percentage of treated women. The difference was significant in the total population (92.3% vs. 50.0%, *p* = 0.007), and HR-HPV subpopulation (90.5% vs. 33.3%, *p* = 0.003). In the HR HPVs-16-18-31 subpopulation, the values were 75.0% and 40.0% (*p* = 0.293). In the total population, 61.5% of treated patients obtained HPV clearance, compared to 50.0% in the control group. Regarding the HR-HPV subpopulation, these values were 66.7% and 44.4%, respectively. Papilocare^®^ demonstrated significant efficacy in repairing low-degree HPV-related cervical lesions and a positive trend to clear HPV in women older than 40 years old in comparison to the watchful waiting approach.

## 1. Introduction

The human papillomavirus (HPV) is one of the most common sexually transmitted infections of the reproductive system [1]. All types of HPV target squamous epithelial cells. The infection starts by viral entry through the traumatized epithelium. Afterward, there are three possible paths: latent infection (there is no gross or microscopic evidence), subclinical infection (there is no clinical disease, but colposcopy or microscopy shows evidence of infection), and clinical disease.

It is estimated that more than 70% of sexually active females will be infected during their lives, and a few will be so more than once. HPV infection is more common among young women [2,3]. Generally, in this population, the infection is asymptomatic and resolves spontaneously without treatment [2].

Nevertheless, the HPV infection sometimes persists, especially in those subtypes classified as high risk (HR), which increases the risk of cervical intraepithelial lesions and cervical cancer [4]. Besides the viral subtype, a time-dependent association of age with HR-HPV clearance has been observed [4,5]. This fact may be associated with the high viral load and viral integration, together with the increased risk of co-infection due to physiological and immunological alterations related to aging. Additionally, the reactivation of previously silenced integration could also influence persistence [6]. Cervical cancer has its maximum prevalence in women between 35 and 44 years. There is also a second lower peak in women aged over 55, which might be associated with waning immunity, reactivation of latent infection, or birth-cohort effects [5]. Furthermore, the mean age of diagnosis is 50 years [7]. 

In this context, Papilocare**^®^** (Procare Health, Barcelona, Spain), a vaginal gel including *Coriolus versicolor* and other ingredients, has proven to be effective and safe in repairing low-grade cervical lesions and enhancing HR-HPV clearance [8,9,10,11,12,13,14,15]. The objective of the present study was to evaluate the efficacy of the vaginal gel in repairing the HPV-dependent low-degree cervical lesions and clearing the HPV in women aged older than 40 years from the PALOMA study (NCT04002154). 

## 2. Materials and Methods

### 2.1. Study Design and Participants

This analysis represents a sub-study of the PALOMA trial, involving specifically women aged over 40 years. Full details regarding the study design, patients, treatment groups, procedures, and analyzed variables have been reported elsewhere [8]. Briefly, women aged between 30 and 65 years presented HPV-related low-grade cytological alterations and concordant colposcopy observations. The study included both low- and HR-HPV cases. The participants were randomized into three scheme groups (1:1:1). Schemes A and B used the vaginal gel once daily for a 21-day treatment/7-day rest therapy (for 1 or 3 months, respectively). This period was followed by an alternate-day therapy for 5 or 3 months, respectively. Scheme C followed a watchful waiting approach. Because evaluating the efficacy of Papilocare**^®^** versus the control approach was the primary objective of the trial, data from treatment schemes A and B were pooled into a single-treatment group for the main analysis.

### 2.2. Data Collection and Evaluated Variables

The primary objective was to evaluate the repair of the cervical-vaginal lesions after 6 months of treatment, defined as normalized cytology and concordant colposcopy observations. Cytology results were classified according to the Bethesda System 2001 [16]. The colposcopy concordance was assessed by the Nomenclature Committee of the International Federation for Cervical Pathology and Colposcopy [17]. A second main objective was to assess the HPV clearance at 6 months, defined as the sum of the total and partial clearance. Total clearance was described as a negative result for the HPV test or the complete disappearance of all strains present at baseline. Partial clearance was defined as the disappearance of at least one strain (compared to baseline) and normalized cytology with concordant colposcopy. 

The sample was analyzed by modified intention to treat, defined as the women included in the study that received at least one dose of the vaginal gel and had the baseline value and one posttreatment value available for the variable. Endpoints were analyzed in 3 groups: total population, HR-HPV subpopulation (strains: 16, 18, 31, 33, 35, 39, 45, 51, 52, 56, 58, 59, 68), and HR-HPV 16-18-31 subpopulation. Since the aim of the sub-analysis was to evaluate the efficacy of the vaginal gel, schemes A and B were pooled into a single-treatment arm.

### 2.3. Data Analysis

Continuous variables were expressed as a mean and standard deviation (SD). Categorical variables were shown as absolute and relative frequencies. Comparisons between groups were analyzed using the χ2 or Fisher’s exact test. Statistical significance was established with *p* < 0.05. The statistical procedures were performed using the SAS 9.4 software.

### 2.4. Data Availability

In accordance with the journal’s guidelines, we will provide our data for the reproducibility of this study in other centers if such is requested.

## 3. Results

Of 91 total women included in the PALOMA trial, 41 patients were older than 40 years. Among them, 31 presented HR oncogenic HPV subtypes, and 14 had HR-HPV 16-18-31 strains. The mean age was 47.7 (SD = 5.6), 46.9 (SD = 5.3), and 46.2 (SD = 5.5), respectively (Table 1). 

### 3.1. Repair of the Cervical-Vaginal Lesions after 6 Months of Treatment

**Figure 1:**
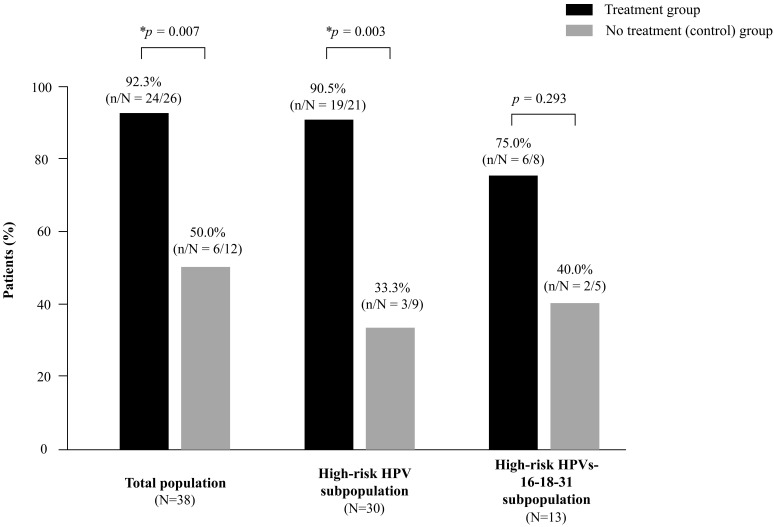
Repair of HPV-dependent low-grade cervical lesions after 6 months of treatment in the total population, HR-HPV and HR-HPV16-18-31 subpopulations pools. Repair of lesions was defined as normalized cytology together with concordant colposcopic observations. * Statistically significant (*p* < 0.05).

After 6 months, normal cytology and concordant colposcopy were achieved by a greater number of women treated with the vaginal gel vs. watchful waiting approach (Figure 1). 

The difference was statistically significant in the total population (92.3% vs. 50.0%, respectively, *p* = 0.007), and HR-HPV subpopulation (90.5% vs. 33.3%, *p* = 0.003). For the HR-HPV 16-18-31 subpopulation, 75.0% achieved repair of the lesions in the treatment group vs. 40.0% in the control group (*p* = 0.293). 

### 3.2. HPV Clearance at 6 Months

There was a greater number of treated women achieving HPV clearance vs. the control group at 6 months. However, the differences were not statistically significant, as shown in Table 2.

## 4. Discussion

### 4.1. Summary of Main Results

Women aged over 40 years are at high risk of developing cancer due to age, the presence of oncogenic strains, and their increased difficulty to spontaneously clearing the viral infection [4,6]. Our data show that the treatment with Papilocare**^®^** for 6 months results in a significantly greater repair of low-grade HPV-dependent cervical lesions and a trend for greater HPV clearance compared to a watchful waiting in women over 40 years.

### 4.2. Results in the Context of Published Literature

Current American Society for Colposcopy and Cervical Pathology (ASCCP) guidelines recommend observation for low-grade squamous intraepithelial lesion (LSIL) or a lesser lesion [18]. This observational management might be long and challenging for both women and their physicians, especially in the case of older patients whose immunosenescence causes the highest persistence rate of HPV (63.6% in ≥55 years) [19]. In fact, this is the primary cause of the development of cervical carcinoma. However, as far as we know, there is no data in the literature exploring the effect of any conservative treatment for low-grade lesions in older women.

The efficacy and safety of the vaginal gel to treat HR-HPV infection in women over 35 years have been observed previously in one independent retrospective observational study conducted by Gajino et al. [13]. They reviewed the clinical history of 86 women who underwent treatment with the vaginal gel for 3 or 6 months. Of them, 79% presented atypical squamous cells of undetermined significance (ASCUS) or LSIL, 89% had HR-HPV, and 84% were treated for 6 months. The mean age was 38.4 years (from 18 to 72 years). After 6 months of treatment, 61% of women aged between 35 and 45 years achieved normalized cytology, and 60% cleared the HPV. Among women aged over 45 years, these figures were 43% and 64%, respectively.

Cortés et al. have recently presented the results of an observational, prospective, non-controlled study called PAPILOBS (clinicalTrials.gov: NCT04199260) in Spain [14,20]. The study included vaccinated or not women over 25 years who tested positive for HPV and with Pap smear of ASCUS or LSIL and concordant colposcopy image. Once the 6 months treatment finished, the patients who still had cytology/colposcopy alterations and/or infection persistency were treated for 6 additional months. A sub-analysis of this observational study has been performed on women aged over 35 and 40 years [21]. It evaluated data of 113 and 74 women, respectively. At 6 months, repaired lesions were observed in 68.7%, and HPV clearance was 60.4% in women aged over 35 years old. These values were 74.0% and 61.1%, respectively, for women aged over 40 years. At 6 or 12 months, in women aged over 35 years, the lesion repair and HPV clearance were 77.9% and 74.1%, respectively. At the same time point, the figures were 82.4% and 75.3%, respectively, for women aged over 40 years. Patients were also stratified according to the presence of HR-HPV [21,22,23], which adds an additional risk factor due to virus persistence. In total, 104 women were included in the younger subgroup and 69 in the older one. Among women aged over 35 years, at 6 months, the repair of the lesions occurred in 68.9%, and the HPV clearance in 58.8%. These data were 73.5% and 59.7%, respectively, in women aged over 40 years. At 6 or 12 months, in the younger subgroup, the figures were 76.9% and 72.8%, respectively. In the case of the older subgroup, the percentages were 81.2% and 73.5%, respectively.

### 4.3. Strengths and Weaknesses

Our study has certain limitations that have been published elsewhere [8]. In this sub-analysis, the main limitation is the small sample size, especially in the subpopulation presenting HR-HPV 16-18-31 strains. Despite the positive trend observed, this limitation complicates obtaining conclusive data regarding clearance. However, significant results were achieved in the repair of lesions in both the total population and HR-HPV subpopulation. Additionally, neither in the original analysis nor in this sub-analysis was a comparison made for low versus high-risk HPV or multiple vs. one-genotype HPV infection.

### 4.4. Implications for Practice and Future Research

Despite the limitations and the need for more data from currently ongoing studies, our findings are in line with previous results in women who are at an additional risk of HPV persistence and with difficulty spontaneously clearing the virus because of their age. Due to these facts, the treatment with the vaginal gel should be considered in clinical practice.

Furthermore, the investigation of the effects of this vaginal gel on low-grade cervical lesions and HPV clearance is being continued in other clinical trials and observational studies (#NCT04210336, #NTC04199078 and #NCT04199260) to consolidate the evidence already shown in the PALOMA study.

## 5. Conclusions

After a 6-month treatment period, the *Coriolus versicolor*-based vaginal gel has demonstrated consistent efficacy in repairing low-degree HPV-related cervical lesions in women older than 40 years. Comparisons with the control group were significant in both the total population and HR-HPV subpopulation. A trend for greater HPV clearance was also observed in these groups.

## Figures and Tables

**Table 1:** Sociodemographic data and patients at follow-up visit at 6 months.

	Total Population	HR-HPV Subpopulation	HR-HPV 16-18-31 Subpopulation
N available	41	31	14
Age, mean (SD)	47.71 (5.56)	46.94 (5.26)	46.21 (5.49)
Follow-up visit at 6 months			
Vaginal gel group, n/N (%)	26/38 (68.4)	21/30 (70.0)	8/13 (61.5)
Control group, n/N (%)	12/38 (31.6)	9/30 (30.0)	5/13 (38.5)

SD, standard deviation. Vaginal gel group: schemes A and B were pooled into a single-treatment arm. Three patients (2 in vaginal gel group and 1 in control group) with missing data at 6 months.

**Table 2:** HPV clearance (sum of total and partial) at follow-up visit at 6 months.

	Total Population	HR-HPV Subpopulation	HR HPV 16-18-31 Subpopulation
Vaginal gel group, n/N (%)	16/26 (61.5)	14/21 (66.7)	5/8 (65.2)
Control group, n/N (%)	6/12 (50.0)	4/9 (44.4)	3/5 (60.0)
*p*	0.725	0.418	1.000

Vaginal gel group: schemes A and B were pooled into a single-treatment arm. *p*: Fisher’s test, statistically significant (*p* < 0.05).

## Data Availability

Data are available upon reasonable request from Javier Cortés (cortes@ocea.es). ORCID-iD: 0000-0002-2372-4066.
